# An integrated double-filtration microfluidic device for isolation, enrichment and quantification of urinary extracellular vesicles for detection of bladder cancer

**DOI:** 10.1038/srep46224

**Published:** 2017-04-24

**Authors:** Li-Guo Liang, Meng-Qi Kong, Sherry Zhou, Ye-Feng Sheng, Ping Wang, Tao Yu, Fatih Inci, Winston Patrick Kuo, Lan-Juan Li, Utkan Demirci, ShuQi Wang

**Affiliations:** 1 State Key Laboratory for Diagnosis and Treatment of Infectious Diseases, First Affiliated Hospital, College of Medicine, Zhejiang University, Hangzhou, Zhejiang Province, 310003, China; 2 Collaborative Innovation Center for Diagnosis and Treatment of Infectious Diseases, Hangzhou, Zhejiang Province, 310003, China; 3 Institute for Translational Medicine, Zhejiang University, Hangzhou, Zhejiang Province, 310029, China; 4 Bio-Acoustic MEMS in Medicine (BAMM) Laboratory, Canary Center at Stanford for Cancer Early Detection, Department of Radiology, Stanford University, School of Medicine, Palo Alto, CA 94304 USA; 5 Department of Urology, First Affiliated Hospital, College of Medicine, Zhejiang University, Hangzhou, Zhejiang Province, 310003, China; 6 Harvard Catalyst-Laboratory for Innovative Translational Technologies, Harvard Medical School, Boston, MA 02115, USA; 7 CloudHealth Genomics, Ltd, Shanghai, 201499, China; 8 Department of Electrical Engineering (By courtesy), Stanford University, Stanford, CA 94305, USA.

## Abstract

Extracellular vesicles (EVs), including exosomes and microvesicles, are present in a variety of bodily fluids, and the concentration of these sub-cellular vesicles and their associated biomarkers (proteins, nucleic acids, and lipids) can be used to aid clinical diagnosis. Although ultracentrifugation is commonly used for isolation of EVs, it is highly time-consuming, labor-intensive and instrument-dependent for both research laboratories and clinical settings. Here, we developed an integrated double-filtration microfluidic device that isolated and enriched EVs with a size range of 30–200 nm from urine, and subsequently quantified the EVs via a microchip ELISA. Our results showed that the concentration of urinary EVs was significantly elevated in bladder cancer patients (n = 16) compared to healthy controls (n = 8). Receiver operating characteristic (ROC) analysis demonstrated that this integrated EV double-filtration device had a sensitivity of 81.3% at a specificity of 90% (16 bladder cancer patients and 8 healthy controls). Thus, this integrated device has great potential to be used in conjunction with urine cytology and cystoscopy to improve clinical diagnosis of bladder cancer in clinics and at point-of-care (POC) settings.

Bladder cancer ranks the second most common malignancy occurring to the genitourinary system^1^,^2^. The incidence of bladder cancer is 20.1 per 100,000 in the US^3^, whereas it is 27 per 100,000 men and 6 per 100,000 women in European Union^4^. Recent statistical data reveal that the incidence of bladder cancer is 80.5 per 100,000 in China^5^. Pathologically, bladder cancer is classified into two groups: superficial tumors (70%) and muscle-invasive (30%) tumors, which often recur after intravesical therapy or require radical cystoprostatectomy^6^. On the other hand, the 5-year survival rate of bladder cancer is closely correlated with clinical staging. For *in situ* and localized bladder cancer, the 5-year survival rate ranges from 70.2–95.9%, and it drops to 5.2–34.5% when bladder cancer becomes regional and distant^7^. Since approximately 80–90% of bladder cancer patients experience only with gross painless hematuria or additionally with frequent urination and urinary urgency, it is of importance to detect bladder cancer at early stages among high-risk populations to avoid radical cystoprostatectomy and to reduce bladder cancer-related mortality.

Currently, urine cytology and cystoscopy are the gold standard methods for collecting laboratory evidence to aid bladder cancer diagnosis. As a non-invasive method, cytological examination is preferably performed on voided urine samples or bladder-washing samples to detect exfoliated cells with pathologically abnormal characteristics. However, this method suffers from low sensitivity and large variations, especially for low-grade tumors. Cystoscopy is, on the other hand, an invasive method to observe tumor lesions on the internal wall of cyst of patients with suspected bladder cancer. However, this method causes significant discomfort, and bladder carcinoma *in situ* may go under-detected^8^. Bladder tumor antigen (BTA) stat and BTA trak tests, which detect urine biomarkers, have shown to report with poor sensitivity and selectivity for the diagnosis of bladder cancer^9^. ImmunoCyt is fluorescence-based cytology with the aid of a cocktail of monoclonal antibodies. It also suffers from low sensitivity (68.3–76.5%) and specificity (62.9–68.5%)^10^. UroVysion is a FISH-based assay for detection of P16 tumor suppressor gene in chromosomes 3, 7, 9 and 17 in exfoliated cells in urine. This assay also has low sensitivity (75.6%) and specificity (84.8%)^11^. Clearly, accurate diagnostic methods are lacking for diagnosis of bladder cancer during early stages for screening.

Recently, studies have shown that EVs or exosomes isolated from biological samples such as plasma, urine, saliva and cerebrospinal fluids can be used for cancer diagnosis and treatment monitoring^12^,^13^. However, the standard method for isolation of EVs (*i.e.* ultracentrifugation) is time-consuming (6–8 h), labor-intensive, and instrument-dependent. Alternative microfluidics-based ExoChips^14^ and Polydimethylsiloxane (PDMS) devices^15^ have been developed for isolation of EVs from serum or plasma. For example, EVs derived from pancreatic cancer patients were captured by CD63 antibody, which was immobilized on ExoChips. The captured EVs were then stained with fluorescence, which revealed that the level of exosomes from the cancer group was significantly higher than healthy individuals^14^. In another study, PDMS devices isolated and enriched EVs from non-small-cell lung cancer patients or ovarian cancer patients using magnetic beads, which were conjugated with a panel of surface biomarkers (*i.e.,* EpCAM, CA125, α-IGF-1R, CD9, CD81 and CD63)^15^. Subsequent chemical lysis of EVs on-chip enabled analysis of intravesicular biomarkers by ELISA, showing that non-small-cell lung cancer patients had a significantly elevated level of IGF-1R than healthy individuals. These studies have clearly demonstrated the feasibility of developing microfluidic devices for isolation, enrichment and analysis of EVs from biological samples derived from cancer patients.

In this manuscript, we developed an integrated double-filtration microfluidic device for isolation, enrichment and quantification of urinary EVs with a size range of 30–200 nm from bladder cancer patients. Based on the principle of size-exclusion, two polycarbonate membranes with a pore sizes of 200 or 30 nm were used to fraction and enrich EVs within this size range. The captured EVs were then analyzed using on-chip ELISA as we previously reported^16^,^17^, which greatly streamlined the process for point-of-care (POC) testing. Our results showed that bladder cancer patients had a significantly elevated level of urinary EVs within the size range of 30–200 nm than healthy controls with a sensitivity of 81.3% at a specificity of 90%. The double-filtration device developed in this proof-of-concept study can be broadly applied to isolate urinary EVs, or EVs with a different size range to screen for genitourinary cancer at the POC.

## Materials and Methods

### Experimental reagents and chemicals

Whatman^®^ Nuclepore™ track-etched membranes with a pore size of 30 or 200 nm were purchased from GE Healthcare Life Science (Shanghai, China). Poly(methyl methacrylate) (PMMA) and double-side adhesive (DSA) were obtained from 3 M Company (St. Paul, Minnesota). 100- and 500-nm fluorescent nanoparticles (FNPs) were purchased from Ocean Nanotech, LLC (Springdale, CA). Biotinylated anti-CD63 antibody, streptavidin-labeled horseradish peroxidase (HRP), and anti-CD9 antibody were obtained from Abcam Inc. (Cambridge, MA). 3,3′,5,5′-Tetramethylbenzidine (TMB), lyophilized bovine serum albumin (BSA), and phosphate buffered saline (PBS, pH 7.0) were purchased from Sangon Biotech Co., Ltd. (Shanghai, China). The Bicinchoninic Acid Kit and 0.22 μm syringe filters were also obtained from Sangon Biotech Co., Ltd. (Shanghai, China).

### Device fabrication

**Figure 1:**
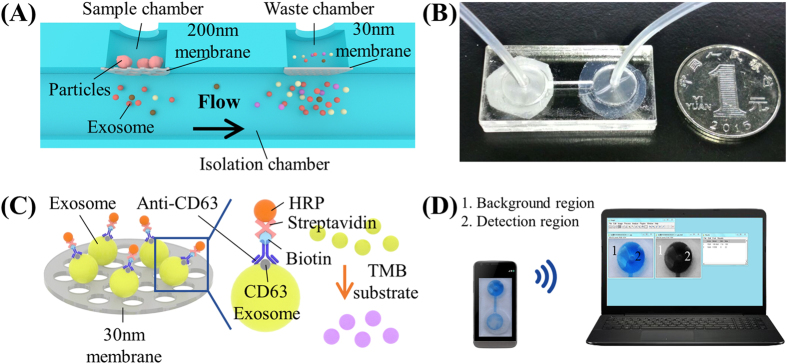
Isolation and detection of EVs from urine using an integrated double-filtration microfluidic device. (**A**) Schematic of a double-filtration microfluidic device for isolation and detection of EVs. Based on size-exclusion, particles larger than 200 nm are excluded by the membrane with a pore size of 200 nm in the sample chamber, whereas particles smaller than 30 nm pass through the double-filtration device. EVs with a size between 30 and 200 nm are isolated and enriched in the isolation chamber. (**B**) Image of an assembled double-filtration device. (**C**) Schematic of direct ELISA for EV detection on-chip. The EVs isolated in the double-filtration device are labeled with biotinylated anti-CD63 antibodies, and then with streptavidin-HRP. Addition of TMB substrate enables blue color development in the double-filtration device. (**D**) The ELISA result is imaged using a smart phone and then transferred to a laptop for data analysis using ImageJ.

**Figure 2:**
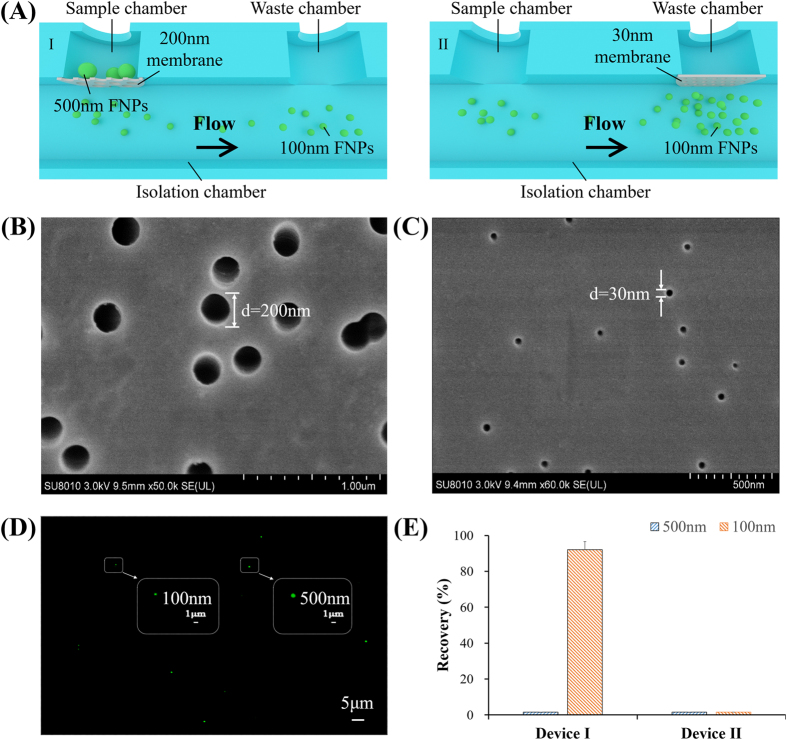
Validation of size-exclusion for double-filtration devices. (**A**) Schematic of Devices I and II to mimic the primary and secondary filtration process of the double-filtration device. A mixture of 500 and 100 nm FNPs was flowed through Device I (contained a 200 nm pore-sized membrane only). The filtrate collected from the waste chamber of Device I was then injected into Device II (contained a 30 nm pore-sized membrane only). The number of 500 and 100 nm FNPs collected from the filtrates was measured with the aid of a fluorescence microscope. (**B**) An scanning electron microscopy (SEM) image of a 200 nm pore-sized membrane (scale bar 1 μm). (**C**) An SEM image of a 30 nm pore-sized membrane (scale bar 1 μm). (**D**) A typical image of 500 nm and 100 nm FNPs. (**E**) Recovery rates of FNPs collected at the waste chambers of Devices I and II.

Devices were assembled with the aid of a laser cutter as previously reported^18^,^19^. The double-filtration device, which had an outer dimension of 20 × 40 × 6 mm, was assembled using four layers of PMMA, four layers of DSA and 2 filter membranes (Fig. 1A). The thickness of four PMMA layers was 2, 1, 1 and 2 mm, respectively. The thickness of DSA was 50 μm. PMMA and DSA were excised using a Universal Laser system (Universal Laser System Inc., Scottsdale, AZ). On the first PMMA layer, two circular openings with a diameter of 1.8 mm were excised to allow for sample injection and waste collection. The second PMMA layer had two separate circular chambers with a diameter of 10 mm. The third PMMA layer consisted of two separate circular chambers with a diameter of 10 mm, which were connected through a microchannel with a width of 1.5 mm. Two Whatman membranes with a pore size of 200 nm and 30 nm were assembled between the second and third PMMA layer (Fig. 1A). There were no openings on the fourth PMMA layer. A total of four layers of DSA were used to assemble the double filtration device, forming two chambers (78.5 μL) above the membranes and two chambers (78.5 μL) below the membranes. Devices I and II (Fig. 2A), which contained a single membrane with a pore size of either 200 nm or 30 nm, were also prepared to validate the double-filtration device.

### Device characterization

To investigate the performance of the double-filtration device, a mixture of 100 and 500 nm FNPs was sequentially injected into Devices I and II using a micropump (Longer Co., Ltd, Baoding, China). 500 μL of the mixture was first injected into Device I containing a single membrane with a pore size of 200 nm, and 500 μL of air was then flowed through the device. The resultant filtrate named Filtrate I was collected from the waste chamber using a tubing with an inner diameter of 0.5 mm. Filtrate I was subsequently injected to Device II, which contained a single membrane with a pore size of 30 nm, and 500 μL of air was flowed through the device. The resultant filtrate named Filtrate II was collected from the waste chamber. Finally, the number of 100 and 500 nm FNPs was counted under a fluorescence microscope (Leica DM4000, German) to verify the precision of size-exclusion of the double-filtration microfluidic device.

### Cell culture

Human bladder cancer cell line T24 was cultured in RPMI 1640 medium supplemented with 10% heat-inactivated fetal bovine serum (FBS), 100 U/mL penicillin, and 100 μg/mL streptomycin (Shanghai, China) at 37 °C in a humid atmosphere with 5% CO_2_. Cell medium was harvested after cell culture for three days, and the number of cells was counted using a hemocytometer (Molecular Devices, San Francesco, CA).

### Urine samples

The use of discarded urine samples was approved by an expedited Institute of Review Board (IRB No. 2016253) at The First Affiliated Hospital, College of Medicine, Zhejiang University. Discarded urine samples (30 mL) were collected from 16 patients who were admitted to the hospital with confirmative diagnosis of bladder cancer on this expedited IRB from which detailed patient information such as disease stage and sex was exempted. No exclusion criteria were specified. As a control, 8 urine samples were collected from healthy subjects and tested. All the urine samples were processed within 6 hours upon collection.

### Isolation of EVs using ultracentrifugation

100 mL of T24 cell culture media was harvested on Day 4. Cells, cell debris and microvesicles (MVs) were removed by centrifuging at 20,000 *g* at room temperature for 15 minutes. The resultant supernatant was filtered through a commercial 0.22 μm filter device to remove potentially contaminated bacteria. The filtrate was then centrifuged at 100,000 *g* at 4 °C for 70 minutes in a type SW32 Ti rotor to precipitate EVs. The crude EV-containing pellets were suspended in 1 mL of PBS after supernatant was carefully removed. To collect EVs from human urine, 100 mL of urine samples was processed as described above. All the isolated EVs were kept at −80 °C until further use.

### Isolation of EVs using double-filtration microfluidic devices

Urine samples and T24 culture media were centrifuged at 20,000 *g* at room temperature for 15 minutes. The resultant supernatant was then filtered through a commercial 0.22 μm filter device to remove potentially contaminated bacteria. 8 mL of the obtained filtrate was injected into a double-filtration device at a flow rate of 40 μL/min and it took approximately 200 min to complete the filtration process. The double-filtration device was then washed with 400 μL of PBS buffer at a flow rate of 40 μL/min for three times. The double-filtration microfluidic device was then flowed with 500 μL of air to completely remove residual liquid.

### Dynamic light scattering (DLS) analysis

Dynamic light scattering analysis of isolated EVs was performed using a Zetasizer Nano S-90 Instrument (Malvern, Worcestershire, UK) at ambient temperature. The detection angle was 90° and the wavelength of helium/neon laser was 633 nm. The size of EVs was determined according to the Stokes–Einstein equation, and the size distribution of EVs was characterized.

### Protein quantification

A bicinchoninic acid (BCA) kit (Sangon Biotech, Shanghai, China) was used to estimate the concentration of EV-associated protein isolated from urine and cell culture media. To establish a quantification curve, 5 μL of BSA standard solutions (0, 0.025, 0.075, 0.125, 0.25, 0.5, 0.75, and 1 mg/mL) was mixed with 5 μL of Solution F in a 96-well plate and then incubated at 37 °C for 30 minutes. 200 μL of BCA working solution was then added to the 96-well plate, which was incubated at 37 °C for 30 minutes. The absorbance was measured at a wavelength of 562 nm using a spectrophotometer (Molecular Devices, San Francesco, CA). 5 μL of EVs, which was isolated from urine or culture media by ultracentrifugation and double-filtration, was tested in parallel with the BSA standards. The concentration of EV-associated protein was calculated by deduction of the amount of protein in the filtrate after double-filtration from the total amount of protein in urine before double-filtration.

### Quantification of EVs using 96-well plate ELISA

The procedure of 96-well plate ELISA included: i) 100 μL of EV suspensions (undiluted, serially diluted to 1:10, 1:100, 1:1, 000, 1:10,000 and 1:100,000) was added to a 96-well plate and then incubated at 4 °C overnight, ii) 200 μL of BSA (1%) was added to each well to exclude unbound active sites at 37 °C for 1 hour, iii) 300 μL of PBS (pH 7.0) buffer was used to wash each well for three times, iv) 100 μL of biotinylated anti-CD63 antibody (1:200, 5 μg/mL) was added to each well and then incubated at 37 °C for 1 hour, v) 300 μL of PBS (pH 7.0) was used to wash each well for three times, vi) 100 μL of streptavidin-labeled HRP (1:2,000) was added to each well and then incubated at 37 °C for 1 hour, vii) 300 μL of PBS (pH 7.0) was used to wash each well for three times, viii) 100 μL of TMB was added to each well and incubated at 37 °C for 10 minutes in darkness, ix) 50 μL of stop solution was then added to each well to terminate further color development, and x) the optical density of each well was measured at a wavelength of 450 nm using a spectrophotometer (Molecular Devices, San Francesco, CA). PBS was used as negative control in 96-well plate ELISA. For quantification, 10 arbitrary units were defined for the colorimetric intensity obtained in the lowest serial dilution (*i.e.,* 1: 100,000 dilution).

### Quantification of EVs using microchip ELISA

The procedure of microchip ELISA for quantification of EVs isolated using a double-filtration device included: i) 300 μL of EV suspensions resulted from ultracentrifugation (serially diluted to 1:10, 1:100, 1:1,000, and 1:10,000) was injected to double-filtration devices, ii) 300 μL of biotinylated anti-CD63 antibodies (1:200) was injected into the double-filtration devices at a flow rate of 40 μL/min and then incubated at 25 °C for 1 hour, iii) 300 μL of PBS was used to wash the devices for three times, iv) 500 μL of air was injected to completely remove liquid, v) 300 μL of streptavidin-labeled HRP (1: 2,000, 0.5 μg/mL) was injected to the devices and incubated at 37 °C for 1 hour in a wet box, vi) the devices were washed as Steps iii and iv, vii) 300 μL of TMB substrate solution was injected into the devices and incubated at 37 °C for 10 minutes in darkness, viii) the development of blue color on chip was imaged using a cell phone (R7, OPPO, Dongguan, China), ix) the images were transferred via wireless communication to a laptop for ImageJ processing, and x) red channel values of blue color as a result of ELISA were used to construct a standard curve as previously reported^16^,^17^. PBS, as negative control, was tested in parallel with EV samples in microchip ELISA.

### Scanning electron microscopy and transmission electron microscopy

The membrane with a pore size of 200 and 30 nm in diameter was observed under a scanning electron microscope (SEM) (Hitachi, Tokyo, Japan). Briefly, membranes were cut into a size of 5 × 5 mm and fixed in 2.5% glutaraldehyde overnight at 4 °C. The membranes were then immersed in PBS (0.1 M, pH7.0) for three times (15 minutes per time). The membranes were subsequently fixed in osmic acid (1%) for 1 hour. The membranes were immersed in PBS for three times as the previous washing step. The membranes were processed with gradient concentrations (30%, 50%, 70%, 80%, 90% and 95%) of acetic acid, and then washed with 100% of ethanol twice (20 minutes per time). The membranes were then treated with the mixture of ethanol and acetic acid (V/V = 1:1) for 30 minutes, followed by pure acetic acid for 1 hour. Images were captured under SEM after critical point drying and gold sputtering.

The EVs, isolated from urine by ultracentrifugation, were visualized using transmission electron microscopy (TEM) (Hitachi, Tokyo, Japan) according to the following steps: 5 μL of EV suspension was fixed in 50 μL of paraformaldehyde (2%), and 2 μL of this mixture was added onto the formvar-carbon coated electron microscopy grids for 3 minutes. 3 μL of osmic acid was then added for negative staining for 1 minute. After the liquid was dried by lens paper, the copper grids were placed under TEM for imaging.

### Clinical testing and statistical analysis

Twenty-four discarded clinical urine samples were obtained from The First Affiliated Hospital, College of Medicine, Zhejiang University (IRB No. 2016253). The concentration of EVs was quantified using microchip ELISA and then log-transformed. Box-Whisker analysis was performed using Origin 8.0 (OriginLab, Massachusetts, USA). Receiver operating characteristic (ROC) curve was plotted for assessment of sensitivity and specificity. A two-sided Student’s t-test was performed using IBM SPSS V22 (New York, US), in which *p* value less than 0.05 was considered statistically significant.

## Results

### Working principle of the integrated double-filtration microfluidic device

To isolate and enrich EVs from urine samples, a double-filtration microfluidic device was developed based on size-exclusion (Fig. 1A,B). Embedded in the device are two membranes with pore sizes of 200 and 30 nm in diameter. According to the working principle of size-exclusion, particles larger than 200 nm are excluded by the membrane with a pore size of 200 nm in the sample chamber, whereas particles smaller than 30 nm pass through the device to the waste chamber. EVs with a size between 30–200 are isolated and enriched in the isolation chamber. Since a larger volume of samples (*e.g.,* 8 mL) can be continuously flowed through the double-filtration microfluidic device, EVs are enriched in the isolation chamber in a smaller volume (*i.e.,* 165 μL). Following isolation and enrichment of EVs in the isolation chamber, EVs are detected using a direct microchip ELISA (Fig. 1C). The isolated EVs are labeled with biotinylated anti-CD63 antibodies, and the resultant immuno-complex subsequently interacts with streptavidin-HRP. Addition of TMB substrate to the device results in blue color development, which is imaged using a smart phone. The images are then wirelessly transferred to a laptop for image processing and data analysis (Fig. 1D).

### Characterization of the double-filtration device

To characterize the double-filtration device, Devices I and II, which mimicked the primary and secondary filtration steps of the double-filtration device, were also assembled, containing a single membrane with 200 or 30 nm pores, respectively (Fig. 2A). The pore size of filtration membranes was approximately 200 nm (Fig. 2B) or 30 nm (Fig. 2C) in diameter confirmed under SEM. A mixture of 100 and 500 nm FNPs was sequentially flowed through Devices I and II at a flow rate of 40 μL/min. A typical image of the mixture of 100 and 500 nm FNPs was shown in Fig. 2D. Figure 2E shows that the recovery of 500 nm FNPs from the filtrate was 0% for both Devices I and II, and that the recovery of 100 nm FNPs from the filtrate was 92.0% and 0% for Devices I and II, respectively. Taken together, these data showed that 500 and 100 nm FNPs were effectively excluded by the filtration membrane with pore sizes of 200 and 30 nm, respectively, indicating that EVs with a size ranging from 30–200 nm could be isolated and enriched by the double-filtration microfluidic device.

### Optimization of flow rates and characterization of EVs

**Figure 3:**
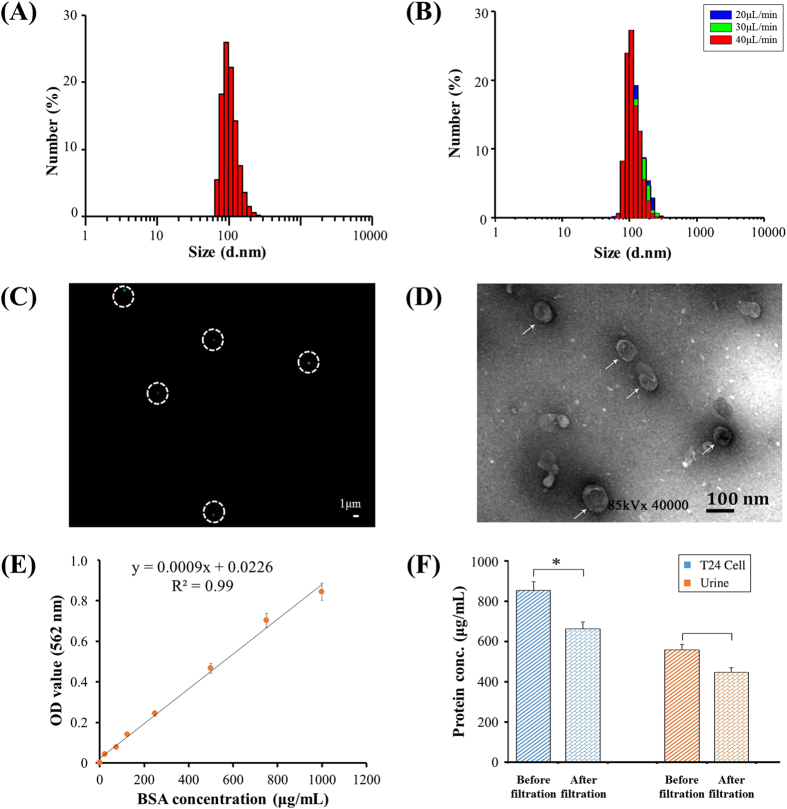
Characterization of EVs isolated from urine using a double-filtration device. (**A**) Size distribution of urinary EVs prior to double filtration. (**B**) Size distribution of urinary EVs after double filtration at a flow rate of 20, 30 or 40 μL/min. (**C**) EVs were stained with Alexa Fluo^®^ 488-labeled anti-CD9 antibody, and then observed under a fluorescence microscope. Scale bar is 1 μm. (**D**) The morphology and size of EVs were observed under TEM. Scale bar is 100 nm. (**E**) Standard curve of the BCA method for protein quantification. (**F**) Quantification of EV-associated protein. EVs isolated from 8 mL of T24 cell culture media or 8 mL of urine collected from a healthy donor using ultracentrifugation were then flowed through double-filtration devices. The concentration of total protein before and after double filtration was quantified using the BCA method. A two-sided Student’s t-test was performed using SPSS V22. The asterisk (*) indicates statistical significance (*p* < 0.05).

To isolate EVs from urine via double filtration, increasing flow rates (*e.g.,* 20, 30, 40 and 50 μL/min) were investigated. Since it was observed that a flow rate of 50 μL/min caused a bulge on the 200 nm pore sized membrane when filtering urine samples, flow rates such as 20, 30, and 40 μL/min were further assessed by checking the intactness of EVs using DLS before and after double-filtration. Prior to double-filtration of urine samples, DLS analysis showed a symmetric unimodal distribution of urinary EVs in size ranging from 68.1 to 295 nm. The average size of EVs after double-filtration was 155 nm and the peak diameter was 105 nm (Fig. 3A). There was no significant difference in peak size of EVs obtained at the flow rate of 20, 30, or 40 μL/min (Fig. 3B). Additionally, no smaller vesicles were detected in the filtrate of urine after double-filtration at the flow rate of 20, 30, or 40 μL/min. The data suggested that EVs maintained their intactness during double-filtration under these flow conditions, and that most EVs within the size range of 30–200 nm passed through the 200 nm membrane and condensed in the isolation chamber. After evaluation and optimization, the flow rate of 40 μL/min was used for isolating EVs from urine using double-filtration microfluidic devices.

EVs isolated from urine were further characterized using fluorescence staining and TEM. Figure 3C shows a typical fluorescence image of EVs labeled with antibody against CD9, which is one of the characteristic protein biomarkers of EVs^20^,^21^. In Fig. 3, the presence of fluorescence-stained EVs was observed as green dots in white circles. The morphology and size of EVs were further investigated with the aid of TEM. EVs indicated by white arrows were close to a circular shape with a diameter of approximately 100 nm (Fig. 3D). In addition, the concentration of protein present in cell media or urine before and after double-filtration was characterized using the BCA method (Fig. 3E). The concentration of protein present in T24 culture media was 853.4 μg/mL, and it was reduced to 662.7 μg/mL after double-filtration; the concentration of protein present in urine was 556.9 μg/mL, and it was reduced to 446.8 μg/mL after double-filtration (Fig. 3F), indicating that the concentration of EV-associated protein was 190.7 μg/mL and 110.1 μg/mL for T24 culture media and urine, respectively. Taken together, the double-filtration microfluidic device demonstrates the capability to isolate EVs from culture media and urine.

### Quantification of EVs using microplate and microchip ELISA

**Figure 4:**
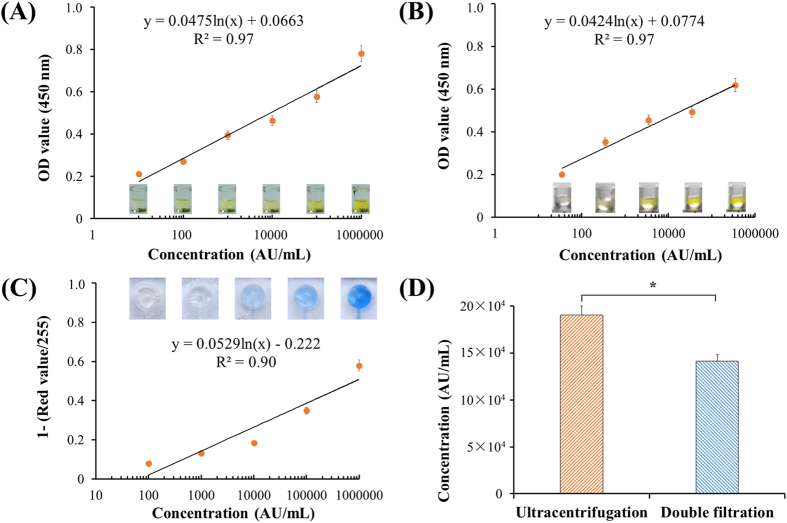
Development of microchip ELISA for detection of EVs. (**A**) Standard curve of microplate ELISA for detection of EVs from cell culture. EVs were isolated from 8 mL of T24 cell media using ultracentrifugation and were 10-fold diluted. The lowest detection point was defined as 10 AU/mL. Side-view images of 96-well microplate ELISA were shown. (**B**) Detection of urinary EVs using microplate ELISA. EVs were isolated from 8 mL of urine from a healthy donor using ultracentrifugation and subsequently quantified using microplate ELISA. (**C**) Standard curve of microchip ELISA for detection of EVs, which were isolated from 8 mL of T24 cell media using ultracentrifugation. Images of microchip ELISA were presented for each standard concentration. (**D**) Comparison of isolation efficiency of EVs between ultracentrifugation and double-filtration. The concentration of EVs was quantified using microchip ELISA. A two-sided Student’s t-test was performed using SPSS V22. The asterisk (*) indicates statistical significance (*p* < 0.05).

To quantify EVs, both microplate and microchip ELISA were developed. The lowest detection point was defined 10 arbitrary unit (AU) of T24 cell culture media, and the standard curve of microplate ELISA was shown as Fig. 4A with R^2^ of 0.97. According to the standard curve, the concentration of EVs from a healthy control’s urine sample was quantified, yielding 34,9818.7 AU/mL. A standard curve was also constructed using this urine sample, with a detection limit of 35.0 AU/mL as shown in Fig. 4B. These results indicate that EVs could be reliably quantified from T24 cell culture media and urine using a direct microplate ELISA. To quantify EVs directly in the double-filtration device, microchip ELISA was also developed with a linearity of 0.90 (R^2^) over the range of 100 to 1,000,000 AU/mL (Fig. 4C). Based on the standard curve of microchip ELISA, the concentration of EVs enriched by ultracentrifugation and double-filtration from 8 mL of T24 culture media was 190,541.5 (AU/mL) and 141,298.7 (AU/mL), respectively. Thus, the isolation and enrichment efficiency of EVs using the double-filtration device was 74.2% compared to ultracentrifugation (Fig. 4D).

### Clinical validation of the double-filtration device

**Figure 5:**
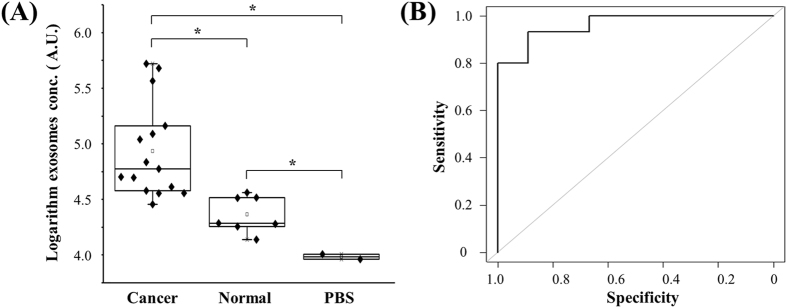
Clinical validation of urinary EVs for differentiating bladder cancer patients from healthy individuals. (**A**) EVs were isolated and enriched from urine samples collected from bladder cancer patients (n = 16) and healthy controls (n = 8), and subsequently tested using microchip ELISA. The log-transformed EV concentrations in bladder cancer patients and healthy controls were compared in a box-plot. A two-sided Student’s t-test was used to analyze the statistical difference between the two groups. The asterisk (*) indicates statistical significance (*p* < 0.05). (**B**) Receiver operating characteristic (ROC) curve was plotted for assessment of sensitivity and specificity. The sensitivity, specificity and the area under ROC curve (AUROC) were analyzed using SPSS V22. The results demonstrated that this integrated EV double-filtration device had a sensitivity of 81.3% at a specificity of 90%.

To validate the double-filtration device, urine samples from bladder cancer patients (n = 16) and normal donors (n = 8) were applied, and the concentration of EVs was measured using microchip ELISA (Fig. 5A). The results showed that the concentration of urinary EVs from bladder cancer patients was in a wide range from 32,790.0 to 368,284.0 (AU/mL), whereas the concentration of urinary EVs from healthy controls was 16,458.4 to 41,279.6 (AU/mL). These results indicate that urinary EVs were successfully isolated using the double-filtration device, and detected by subsequent microchip ELISA. More importantly, it was found that the concentration of urinary EVs from bladder cancer was significantly higher than that from healthy controls (Fig. 5A). In addition, a receiver operating characteristic (ROC) curve was plotted for clinical validation. As shown in Fig. 5B, the microchip ELISA had a sensitivity of 81.3% at the specificity set to 90.0% for identifying bladder cancer patients from healthy controls, and the AUROC was 0.96.

## Discussion

In this study, we developed an integrated double-filtration microfluidic device, which isolates, enriches and quantifies urinary EVs on-chip to assist in screening for bladder cancer at the POC (Fig. 1). The developed double-filtration device was based on size-exclusion to isolate EVs with size ranging from 30 to 200 nm. For device characterization, a mixture of 500 and 100 nm FNPs was sequentially flowed through Devices I and II containing 200 and 30 nm pore-sized membranes, which mimicked the primary and secondary modules of the double-filtration device (Fig. 2A). The results clearly showed that 500 nm FNPs were excluded by the 200-nm pore-sized membrane and that 100 nm FNPs were retained by the 30 nm pore-sized membrane (Fig. 2E), indicating that EVs can be isolated and enriched by setting up size thresholds between 30 and 200 nm. Indeed, fluorescence staining successfully detected one of the hallmark molecules (*i.e.,* CD9) of EVs isolated from urine samples and T24 cell culture media using the double-filtration device based on size-exclusion (Fig. 3C). Furthermore, the morphology of EVs isolated from urine or cell culture media showed round or ellipsoidal particles with a size ranging from 40–100 nm (Fig. 3D), confirming that the double-filtration device based on size-exclusion can be reliably used to isolate and enrich EVs from urine or cell culture media.

The double-filtration microchip approach recovered 74.2% of EVs compared to ultracentrifugation, which is moderately lower than a previous study based on a sequential filtration strategy for isolating EVs (81%) with the size of 40–100 from cell culture^22^. The lower isolation efficiency of double filtration compared to ultracentrifugation may result from the following possible reasons or their combination. The, impurities such as larger extracellular vehicles may attribute to the overestimation of EVs isolated by ultracentrifugation, which is based on the gravity difference among EVs after multiple rounds of centrifugation at varying speeds. The presence of EVs with a size larger than 200 nm would contribute the overestimation of EVs isolated from ultracentrifugation by ELISA, which in turn would lower the isolation efficiency of EVs using double-filtration. Although EVs, which are nanoscale particles with bilipid membranes, may be squeezed and broken into undetectable particles during the double-filtration process, DLS analysis of the filtrate after double filtration did not detect particles. Additionally, no smaller vesicles were detected in the filtrate of urine after double-filtration at the flow rate of 20, 30, or 40 μL/min (Fig. S1). Thus, the moderately lower recovery efficiency of EVs from double filtration is mostly likely due to the presence of impurities in EVs precipitated by ultracentrifugation.

Tamm-Horsfall protein (THP), as an abundant protein in human urine, may affect the isolation and detection of EVs in this double-filtration based microchip ELISA. In an earlier study, urine samples were treated with a reducing agent, dithiothreitol (DTT), which was followed by re-ultracentrifugation to remove abundant THP from urine for analyzing low-abundance proteins^23^. However, the use of DTT to denature THP is also of a concern, since it can also denature other proteins including active protein biomarkers on the outer surface of EV membranes^24^. Thus, DTT was excluded from urine treatment for profiling protein biomarkers from urinary EVs by mass spectrophotometry^24^. Similarly, DTT was not used to remove THP from urine, since surface biomarker CD63-based ELISA was utilized for quantification of urinary EVs. In addition, THP is mainly secreted by the loop of Henle at the thick ascending limb in kidney^25^,^26^ and forms into polymeric filaments in urine^27^. Although polymeric THP filaments may trap some EVs, these trapped EVs would be more likely derived from renal corpuscles or secreted by kidney. Since this portion of EVs would less likely add clinically relevant diagnostic values for assessing the risks of bladder cancer, we excluded the use of DTT to remove THP from urine in the CD63-based microchip ELISA for analyzing urinary EVs derived from bladder cancer patients.

In this study, we aimed at developing an integrated device for isolating and quantification of EVs from urine for initial screening of bladder cancer at the POC. Coupling on-chip ELISA with isolation and enrichment of EVs in an inexpensive, disposable device significantly streamline the analysis of urinary EVs at the POC. More importantly, the concentration of EVs from bladder cancer patients was significantly elevated compared to healthy controls, and microchip ELISA differentiated bladder cancer patients from healthy controls with a sensitivity of 81.3% at a specificity of 90% (Fig. 5). Although bladder cancer is currently diagnosed using cytology in combination with cystoscopy and histology, they are limited by invasive examination and poor sensitivity. Non-invasive approaches to assessing urinary biomarkers of bladder cancer (*e.g.,* NMP22, BTA and urinary surviving) generally lack sensitivity for low-grade bladder cancer, and these urinary biomarkers may be falsely elevated due to non-malignant conditions and hematuria^28^. The presented double-filtration device for analyzing the concentration of urinary EVs represents an attractive approach to aid initial screening of bladder cancer at the POC.

From a perspective of POC testing, the developed EV quantification modality is advantageous over traditional methods for isolating and detecting EVs from urine samples, considering insufficient infrastructure and inexperience operators in POC settings^29^,^30^. The double-filtration device eliminated the need for an ultracentrifuge to isolate EVs from urine, which is costly and time-consuming. The principle of cell phone-based on-chip ELISA, which we previously developed^16^,^17^, was used in this study to accommodate convenient detection/quantification of urinary EVs without reference to a spectrophotometer. Compared to other existing EV analytical methods such as ExoChips and immunoisolation-based devices, our integrated EV analytical device is more cost-effective. Since ExoChip relies on the use of antibodies immobilized on the surface of microchannels to capture EVs from serum, it is costly because of the use of large amount of antibodies, and it may suffer from extensive assessment of flow rate to achieve optimal sensitivity and specificity. In contrast, our double-filtration device is based on size-exclusion, which eliminates the use of capture antibody to isolate EVs and obviates the tedious optimization of flow rate. Immunoisolation-based devices, albeit of shorter assay time (*e.g.,* 1.5 h), requires sophisticated analytical tools such as upright epi-fluorescence microscope to analyze the plasma-derived exosomes from non-small-cell lung cancer patients, which is not practical or user-friendly for POC testing. Thus, our integrated, inexpensive, and disposable microfluidic device is advantageous over current exosome and EV analytical methods for POC testing.

## Conclusion

In conclusion, we developed a double-filtration device for isolation and enrichment of EVs from urine and subsequent detection via microchip ELISA with the aid of a cell phone. This integrated approach demonstrated the proof-of-concept of using the concentration of EVs to differentiate bladder cancer from healthy controls in a non-invasive manner. In this approach, EVs are isolated and enriched based on size-exclusion by membranes, eliminating the need for immuno-capture on-chip and significantly simplifying microchip ELISA at the POC. Furthermore, this platform and cell phone system have potential to be integrated with advance sensor technologies, including surface plasmon resonance tools, nanomechanical platforms, and electrical sensing systems^31^,^32^,^33^. Nevertheless, future studies will benefit from clinical validation in a larger population and applications in other genitourinary cancers.

## Additional Information

**How to cite this article:** Liang, L.-G. *et al*. An integrated double-filtration microfluidic device for isolation, enrichment and quantification of urinary extracellular vesicles for detection of bladder cancer. *Sci. Rep.* **7**, 46224; doi: 10.1038/srep46224 (2017).

**Publisher's note:** Springer Nature remains neutral with regard to jurisdictional claims in published maps and institutional affiliations.
